# MRI-based brain stroke classification using a hybrid vision transformer–BiLSTM architecture

**DOI:** 10.3389/fneur.2026.1802972

**Published:** 2026-04-10

**Authors:** Reeta Samuel, Thanapal Pandi

**Affiliations:** School of Computer Science Engineering and Information Systems, Vellore Institute of Technology, Vellore, Tamil Nadu, India

**Keywords:** BiLSTM, brain stroke, convolutional neural network, MRI, ViT

## Abstract

**Introduction:**

Stroke is a prominent cause of long-term disability, impacting patients’ socioeconomic status in daily life. Hemorrhagic and ischemic strokes differ in dimensions, forms, and locations, posing challenges for automated detection. Magnetic resonance imaging (MRI), particularly diffusion-weighted imaging (DWI), reveals changes in fluid balance, thereby enabling early detection. Hence, MRI scans are more accurate than computed tomography (CT) scans due to their increased sensitivity.

**Methods:**

To categorize brain strokes, a hybrid model combining bidirectional long short-term memory (BiLSTM) with a vision transformer (ViT) was developed using an MRI dataset from a private source. ViT identifies qualities using MRI. The ViT captures global contextual and spatial representations using patch-based self-attention (16×16 patches, 256-dimensional projections, four transformer encoder layers with eight attention heads), whereas the BiLSTM network (128 and 64 units) models dependencies inside transformer-encoded features. A comparative study was conducted for the hybrid architecture with deep learning models, including a convolutional neural network (baseline, 85.5%), VGG16 (87.8%), ResNet50 (89.2%), ViT (91.3%), and BiLSTM (88.6%).

**Results:**

The hybrid ViT–Bi-LSTM model achieved a precision of 97.35%, recall of 93.04%, accuracy of 95.21%, F1-score of 95.15%, and ROC-AUC of 99.36%, outperforming other comparative approaches. The standalone ViT achieved an accuracy of 91.3%, exceeding the CNN-based methods. In 5-fold cross-validation, the hybrid ViT-BiLSTM model achieved an average accuracy of 96.61%, with a standard deviation of 0.78, indicating stable performance across folds. These findings validate the combination of bidirectional temporal modeling with transformer-based feature extraction.

**Conclusion:**

By capturing the global spatial context through self-attention and bi-directional features via recurrent processing, ViT with Bi-LSTM networks expands stroke classification from MRI data. The ViT–Bi-LSTM model showed a promising approach for clinical decision support systems in early stroke diagnosis. Future research will use federated learning (FL) to protect privacy and assess model generalizability across multi-institutional MRI datasets.

## 1 Introduction

Stroke is a medical condition that can affect people of nearly all ages, with a higher prevalence rate among people over 65 years of age. There are two primary categories of stroke: ischemic and hemorrhagic. An ischemic stroke occurs when the brain vessels become blocked, whereas a hemorrhagic stroke results in the rupture of blood vessels inside or surrounding the head (1). Brain stroke is a restorative condition that develops when the blood flow to a particular region of the brain is disrupted or reduced, resulting in the loss of nearby brain cells. Analyzing a stroke after its occurrence is not a useful approach (2). In many developed countries, stroke is among the main causes of long-term impairment. Approximately 16 million people worldwide are affected by stroke annually. This disorder comprises a kind of illness depicted by unexpected, specified interruption of blood flow to the brain, causing nervous indications that last for above 24 h. Brain ischemia is caused by embolus or thrombus obstruction, which causes ischemic strokes. In contrast, hemorrhagic stroke occurs when an injured blood vessel bursts and bleeds into brain tissue, typically affecting increased intracranial pressure (3).

Analyzing stroke concern includes collecting a detailed medical record for neurological and substantial assessment, and presenting brain tomography tests, such as MRI or CT scans. These phases help rule out other disorders that mimic strokes and establish the type, position, and boundary of the brain injury (4). Pre-emptive schemes for diagnosing ischemic stroke threat are envisioned to decrease or prevent the incidence of medical periods with premature death correlated with ischemic stroke. Over the past few years, several computerized techniques and implementations have emerged for the quick identification of brain illnesses. Artificial intelligence (AI) and deep learning (DL) have been used to construct accurate and automatic results for stroke diagnosis (5). AI models have been used to infer facial imaging data and have attained numerous positive outcomes (6). Recently, the role of deep learning (DL) and machine learning (ML) have expanded drastically in different medical treatments. These DL- and ML-based methodologies are cutting-edge tools that can aid clinical specialists in making informed decisions and predictions (7).

Over the past few decades, numerous studies have focused on applying DL and ML to stroke recognition, resulting in significant improvements in speed and accuracy. Among the state-of-the-art architectures, the vision transformer (ViT) (8) has gained a reputation as a powerful model that develops the transformer outline to efficiently identify the background and extract features from raw image data. This represents a significant deviation from traditional convolutional neural networks (CNNs) (9, 10), which mostly emphasize localized image regions. With the adoption of the ViT architecture, the classification of brain strokes—which has traditionally been based on handcrafted features and conventional ML approaches—has seen significant advancements. These models make more specific and efficient diagnoses possible by producing expressive representations from medical imaging data. They have the ability to obtain easy-to-read images. ViT models identify subtle patterns essential for detecting strokes by analyzing contextual correlations across image segments.

Deep learning techniques, especially convolutional neural networks (CNNs), have been explored for their ability to detect Alzheimer’s disease. Deep super-resolution generative adversarial networks (DSR-GANs) and CNNs have shown promising result in improving accuracy of Alzheimer’s disease diagnosis (11). Deep learning has revolutionized medical imaging by extracting features from data, and it has proven effective in identifying Alzheimer’s disease (AD) using positron emission tomography (PET), magnetic resonance imaging (MRI), and computed tomography (CT) scans (12).

**Figure 1:**
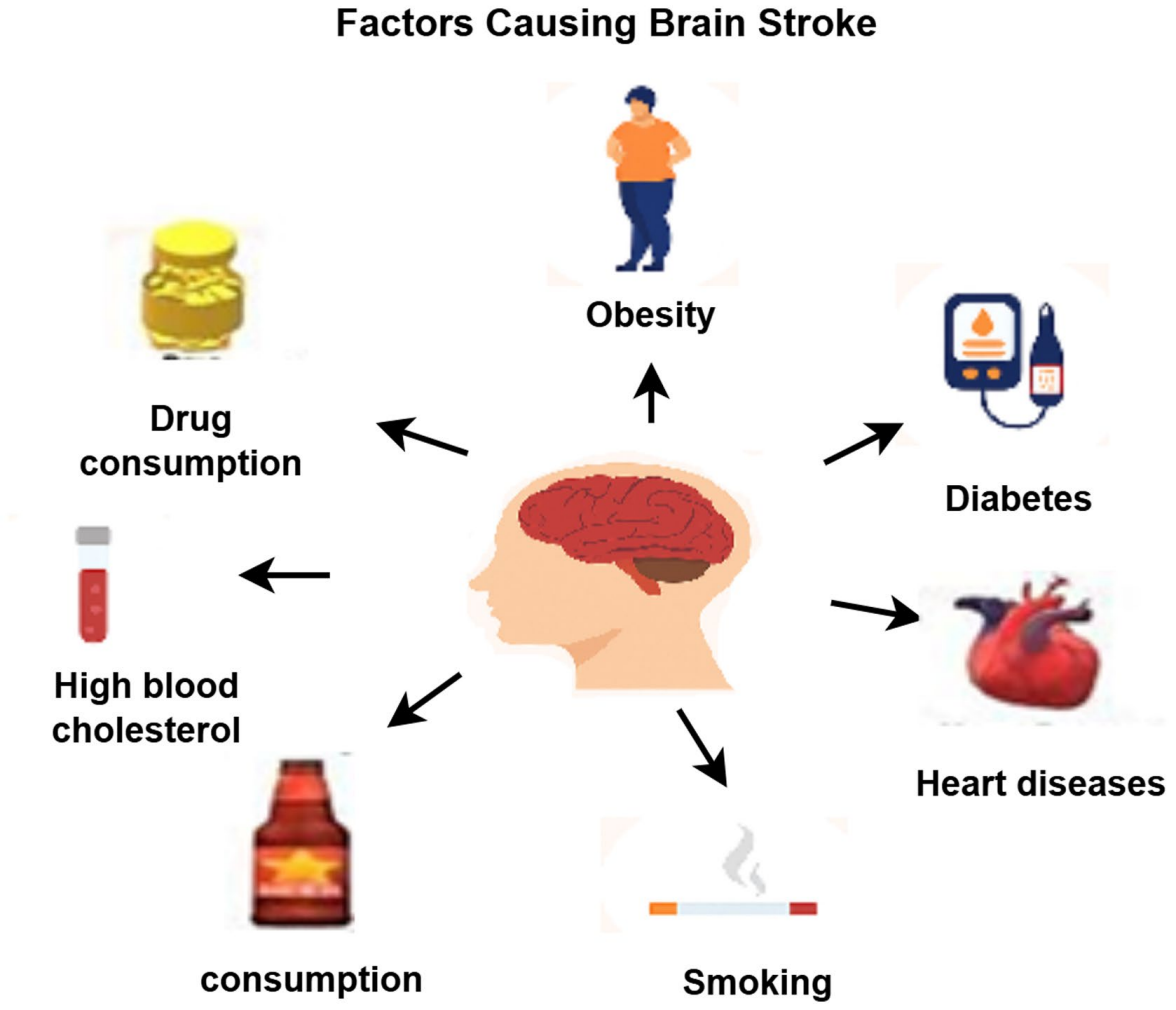
Aspects that affect brain stroke.

This study presents a deep-learning-based framework for predicting acute stroke from MRI scans. By employing effective image pre-processing and a robust hybrid learning architecture, the proposed method aims to improve the accuracy of early stroke detection and classification. In the pre-processing stage, a median filtering method was used to reduce noise and increase image clarity. A hybrid Vision Transformer and Bidirectional Long Short-Term Memory model is used to classify brain strokes; the ViT captures spatial characteristics via self-attention, and the BiLSTM network captures temporal dependencies and contextual relationships within feature sequences. The BiLSTM network model uses sequential dependencies in the extracted feature representations, thereby producing a more precise and discriminative classification, whereas the ViT component successfully captures global and contextual spatial characteristics from MRI scans. Using private brain stroke MRI data acquired from the KC Hospital, the efficacy and dependability of the proposed framework were confirmed, and its performance was assessed using several common evaluation measures. Cerebrovascular diseases, also known as stroke, occur when blood flow to the brain is cut off. Figure 1 shows the main risk factors for these diseases. The picture indicates that stroke risk is affected by a wide range of lifestyle choices and medical conditions, such as obesity, diabetes, high blood pressure, heart disease, smoking, alcohol consumption, high cholesterol, and drug abuse. These elements stimulate thrombus development, increase blood pressure, and consequently impede oxygen distribution to the cerebral tissues by damaging vascular health. Developing successful preventive plans and prompt clinical treatments depends on an awareness of these influencing elements. Consideration of these contributing factors is crucial for advancing effective prevention strategies and timely clinical interventions. The key contributions of the proposed approach are as follows:

- A private dataset was gathered and pre-processed using a distinctive digital imaging and communications in medicine (DICOM) brain stroke magnetic resonance imaging (MRI) dataset. This dataset, obtained from a hospital, was subsequently converted into the JPEG format and served as a valuable resource for future research and development.
- Median filtering was applied during the pre-processing stage to suppress noise and enhance image quality before classification.
- Development of an innovative ViT–BiLSTM hybrid model that combines transformer-based global spatial feature extraction (patch-based self-attention with 16 × 16 patches) with bidirectional temporal modeling (128 and 64-unit BiLSTM) for enhanced brain stroke classification from MRI images.
- The framework shows promise for aiding clinical stroke diagnosis by enhancing classification reliability using real-world MRI data. We used ViT-BiLSTM to interpret the final models and gain insights into how the model processes brain stroke images.
- To prevent overfitting and ensure stability, we employed dropout, normalization techniques (batch and layer), and 5-fold cross-validation. Training was optimized using early stopping and learning rate adjustments, and the final model was evaluated using accuracy, precision, recall, F1-score, and ROC/PR curves.

## 2 Related works

Bhandari et al. developed a web-based stroke risk assessment tool that uses a strong machine learning standard and a unique sequence to enhance the recognition of stroke risk factors associated with current treatments through logistic regression and SMOTE oversampling. Early detection of individuals at high risk of stroke may substantially improve control outcomes (13).

Alsieni et al. proposed a model directing brain strokes, which primarily modifies entities in people over 65 years of age and can be either ischemic or hemorrhagic. Rapid analysis and treatment are necessary; however, access to expanded imaging performance is often limited to obtaining areas. This study introduces the Enhanced Brain Stroke Detection and Classification employing Artificial Intelligence with Feature Fusion Technologies (EBSDC-AIFFT) model, which affects medical images to achieve better analytical accuracy: The model uses the Inception-ResNet-v2 framework, convolutional block attention module-ResNet18 approach, multi-axis vision transformer method, and variational autoencoder model (1).

Ahed Abugabah et al. hosted a simplified deep learning outline conceived for the effective and perfect classification of brain tumors and stroke lesions on devices with reduced properties. This framework employs MobileNetV3 for feature extraction and combines a hybrid optimization algorithm that syndicates the bat algorithm with differential evolution. To decrease memory management and computational challenges, the model utilizes quantization techniques and compressed medical image information. The outline achieved classification accuracies of 96.3 and 94.8% for brain tumor and stroke lesion detection, respectively, surpassing those of the current running techniques (14).

Shirodkar et al. proposed a system for brain computer interfaces (BCIs) that exploits brain signals to demonstrate motor imagery (MI). Electroencephalogram (EEG) signals are critical in this framework; however, classifying these signals in patients with stroke appears to present challenges because of the variability in patterns. The recommended system combines feature extraction with progression optimization to develop MI classification. It works by using event-related desynchronization (ERD) to select frequency bands that are distinctive to entities, thereby guaranteeing permanent signal acquisition. Longitudinal and time-based features were obtained using a scale-invariant feature transform (SIFT) and a one-dimensional convolutional neural network (1D CNN) to capture EEG changes. The system combines these features using an enhanced extreme learning machine (EELM), with hidden layer weights optimized through differential evolution (DE), particle swarm optimization (PSO), and dynamic multi-swarm particle swarm optimization (DMS-PSO). Testing on 50 patients with stroke demonstrated a 97% accuracy rate with DMS-PSO and a 10-fold cross endorsement. Additionally, tests on the BCI Competition IV datasets produced 95% accuracy on Dataset 1a and 91.56% on Dataset 2a, thereby substantiating the efficacy of the proposed system (15).

Arman et al. introduced a method for identifying intracranial hemorrhage, which involves bleeding within the skull or brain tissue and is classified into five categories: epidural, subdural, subarachnoid, intraparenchymal, and intraventricular. Although the timely discovery of internal hemorrhage is critical, a lack of radiologists can cause diagnostic delays. We conceived a computerized system to reveal intracranial hemorrhage from CT scans by applying an optimized densely connected convolutional network (DenseNet) enhanced with Bayesian optimization (BO). BO was engaged to establish the best learning rate, optimizer, and quantity of nodes in the deep layers. Our model not only detected the existence of hemorrhage but also differentiated between its subtypes. The optimized DenseNet exhibited remarkable functioning and will aid surgeons in composing learned outcomes to improve patient care (16).

Hossain et al. have highlighted recent research that concentrates on using deep learning for automated stroke diagnosis through brain imaging techniques, especially CT scans, to address the challenges of manual interpretation. To boost diagnostic accuracy, hybrid models that combine vision transformers (ViTs) with long short-term memory (LSTM) networks have been developed. These representations effectively detect global spatial features using transformer-based self-attention and sequential dependencies through recurrent learning. To improve strength and clinical relevance, these systems often challenge class imbalance and participate in explainable artificial intelligence (XAI) tools, such as similar attention maps, SHAP, and LIME, which enable interpretable and consistent estimates. Experimental results from both private clinical and public benchmark datasets have disclosed that ViT–LSTM-based models consistently surpass traditional convolutional neural networks (CNNs) and standalone transformer models, showcasing superior accuracy, generalizability, and potential for supportive clinical verdicts in stroke analysis (17). Table 1 presents an outline of research directed toward the detection and classification of strokes.

**Table 1:** Summary of research on stroke detection and classification.

Author	Year	Objective	Method	Dataset	Result
Alsieni and Alyoubi (1)	2025	To create an improved system for detecting and classifying brain strokes using biomedical imaging, and enhancing diagnostic precision.	EBSDC-AIFFT model →Pre-processing (resizing, normalization, augmentation) + Feature Fusion (Inception-ResNet-v2, CBAM-ResNet18, MaxViT) + Classification (Variational Autoencoder)	Brain Stroke CT Image Dataset (Kaggle, 2,501 images: 1551 normal, 950 stroke)	Accuracy: 99.09%, outperforming existing models
Alrowais et al. (3)	2025	Brain stroke detection and classification using facial imaging with deep transfer learning	ENDDFTL-ABSPFI model → Pre-processing (Fuzzy Filter) + Feature Extraction (Inception-V3, EfficientNet-B0) + Classification (CNN-BiLSTM) + MOSFO	Kaggle Facial Image Dataset	Accuracy: 98.60% (superior to existing methods)
Alomoush et al. (23)	2025	Enhancing brain stroke prediction through an improved meta-heuristic algorithm	mMGO (Modified Mountain Gazelle Optimizer with Joint Opposite Selection) + KNN classifier	Kaggle Brain Stroke Dataset + CEC 2020 benchmark functions	Accuracy: 95.5%, Sensitivity: 99.34%, Specificity: 98.99%, Precision: 99.21%
Hossain et al. (17)	2025	Create an interpretable deep learning model for identifying stroke features from CT scans, addressing interpretation, imbalance, and transparency while ensuring reliable clinical decisions.	Hybrid Deep learning model: Vision Transformer (ViT) + Long short-term memory (LSTM) + Explainable AI methods, SGD, RMSProp, Adam, AdamW used.	BrSCTHD -2023 dataset, Rajshahi Medical College Hospital dataset.	Accuracy:96.61 \%, outperformed traditional CNN-based and ViT models.
Cai et al. (24)	2024	Multimodal AI for mobile stroke triage	DL, Audio-Visual + Mobile Edge Computing	269 Patient Records	Accuracy: 80.85%, Sensitivity: 90.63%
Kina et al. (25)	2025	Rapid MRI-based stroke detection	Efficient Net + Squeeze Attention, SMOTE, Grad-CAM	MRI datasets (balanced via SMOTE)	High efficiency & explainability
Mena et al. (26)	2024	Stroke lesion segmentation & classification	CLCI-Net + DL + ML	MRI Brain Images	DL: 84%, ML: 95%
Gnanabaskaran et al. (27)	2025	Early cerebral stroke detection	Pre-trained VGG16 + SVM	Annotated Brain Scans	Accuracy: 96.5%
Leng et al. (28)	2024	To enhance MI-based BCI performance for stroke rehabilitation by improving EEG analysis	Modified S-Transform (MST) for time-frequency analysis + Dense Graph Convolutional Network (DenseGCN) for deep EEG feature learning	EEG signals from stroke patients (MI-BCI experiments)	Peak Accuracy: 90.22%, Avg. ITR: 68.52 bits/min
Pokorny et al. (29)	2024	To systematically evaluate and optimize SVM configurations for microwave-based brain stroke classification	Support Vector Machine (SVM) with Principal Component Analysis (PCA) for dimensionality reduction	Synthetic datasets generated from 3D numerical human ear models with varying stroke sizes, positions, and dielectric properties (antenna array sys-term)	Best-case classification accuracy ≈ 70% for ischemic stroke detection under high-variability settings
Mandhare and Kshirsagar (30)	2026	To develop an optimized deep learning model for accurate and early stroke detection using CT brain images	EHTO_DKN model → Pre-processing (Double Bilateral Filter for denoising, MAD-Net for skull segmentation, augmentation) + Feature Extraction (shape, statistical, LIFT + DCT) + Classification (Deep Kronecker Network with Elk Herd Taylor Optimizer)	CT Brain Image Dataset	Accuracy: 90.687%, Sensitivity: 89.696%, Specificity: 91.445%
Wang et al. (31)	2025	Cognitive & motor impairment classification in stroke	Radiomics + 14 ML Models + SHAP	3D MRI (OAx T2 Propeller)	Cognitive: 92%, Motor: 82.5%
Hassan et al. (32)	2024	To develop a novel compressive sensing (CS) technique for efficient compression and reconstruction of multi-lead ECG signals	Dynamic Compressive Sensing (CS) with an adaptively constructed sensing matrix derived from the fusion of multiple ECG leads	ECG signals from healthy individuals and patients with bundle branch block, cardiomyopathy, and myocardial infarction (6 standard leads evaluated)	Achieved a compression ratio (CR) of 16 with PRD < 8%; improved signal reconstruction quality compared to recent CS methods; better performance using all 6 leads

Mohsen et al. proposed integrating wearables with deep learning to monitor strokes in smart hospitals via a web application. Three DL models - LSTM, GRU, and BiLSTM - were evaluated using gender and health status. The BiLSTM achieved the highest accuracy of 100%, versus the LSTM’s 99.90% and the GRU’s 99.80%, helping physicians diagnose strokes efficiently (18). Saeed Mohsen et al. worked on the use of AI for the classification of EEG signals between epileptic and non-epileptic areas. This study discusses the use of LSTM and support vector machine (SVM) classifiers for EEG data, specifically the Bonn data set. Using K-fold cross-validation and Walsh-Hadamard transform, the accuracy of the LSTM classifier was 99.00%, while the SVM classifier had an accuracy of 91%. The SVM classifier had a sensitivity of 93.52% and a specificity of 91.3%. The LSTM classifier takes 2000s for training and 2,500 s for testing (19). In their study, Mohsen et al. used AI and magnetic resonance imaging (MRI) for brain cancer recognition. The authors used a convolutional neural network (CNN) model that identified meningiomas and pituitary tumors using 1,800 MRI images. The CNN model, implemented using TensorFlow and Python, had a weighted precision of 95.82% and an accuracy of 95.78% for brain tumor detection (20).

## 3 Proposed methodology

This section outlines the key stages of the proposed method to facilitate a comprehensive evaluation. The initial focus for examination was a dataset. Subsequently, processing techniques were applied to ensure data uniformity; median filters were used during the pre-processing step to enhance the picture quality in this study. Initially, DICOM files were converted to the JPG format and subsequently split into three separate groups: training, testing, and validation by assigning a stroke label to every image. Combining ViT and BiLSTM, a hybrid design was developed to analyze MRI images and categorize brain strokes. To capture sequential dependencies and reflect the link between several spots inside an image, a spatial feature learning model and a global context model were combined. Each input image was first segmented into patches and then flattened to reproduce the spatial characteristics of the brain images. Linear projection was used to project each patch onto a higher-dimensional latent feature space.

**Figure 2:**
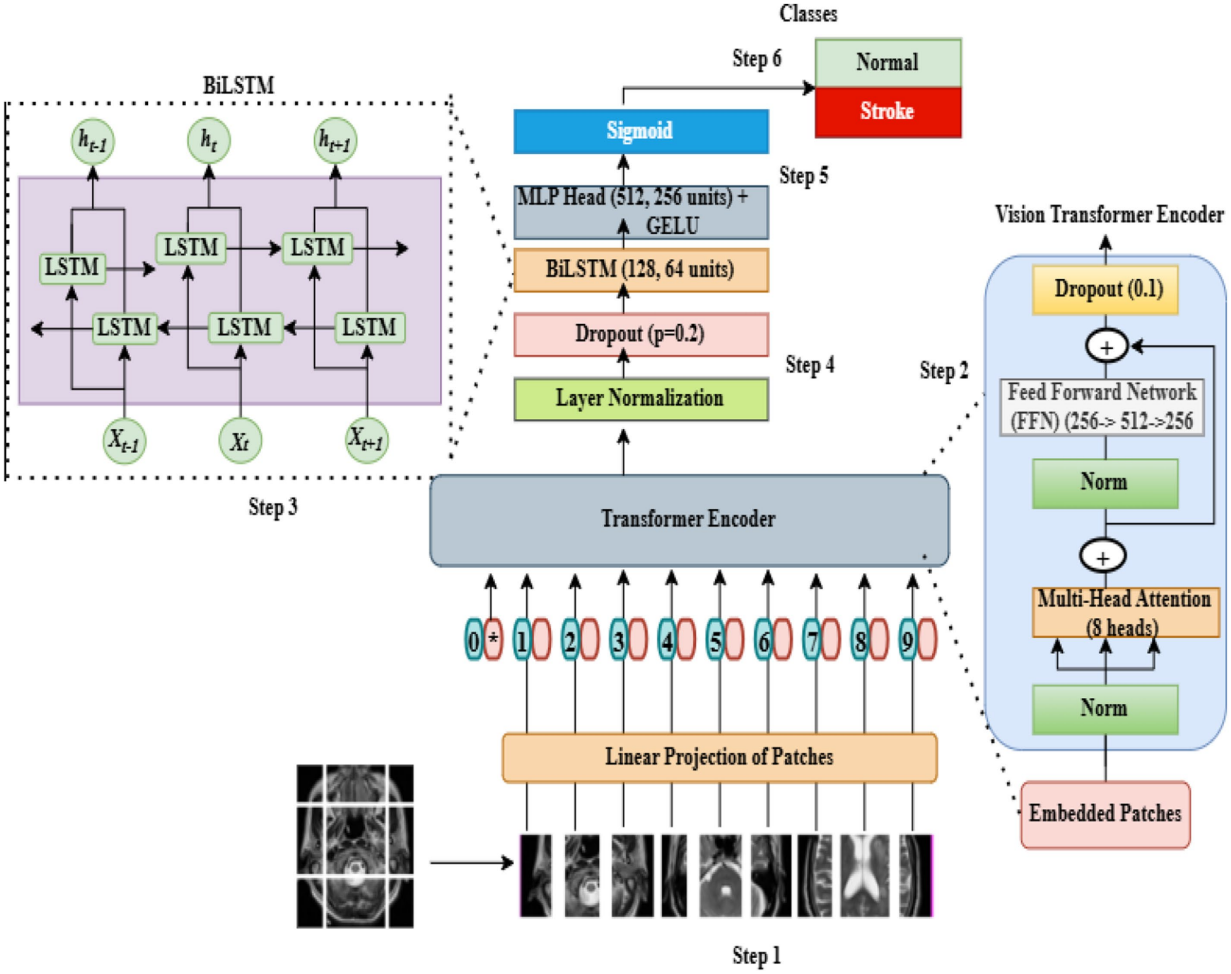
Framework of the proposed hybrid ViT-BiLSTM model.

To preserve the positional information of patches relative to other patches, positional embedding matrices were generated, and the embedded matrices were input into the ViT encoder. The ViT architecture utilizes multiple heads of positional embedding to incorporate various patches of an image. It produces an output matrix that encapsulates the global characteristics of the entire image, as well as the long-range positional relationships that exist among the patches. The output of the ViT encoder was then passed to a bidirectional long short-term memory (BiLSTM) layer that processes the input data both forward and backward and is capable of building an understanding of the temporal context of both past and future developments related to a given patch of an image. The output of the BiLSTM layer is further refined using a multi-layer perceptron (MLP) with gelu activation, whereas a final output layer uses sigmoid activation to differentiate between stroke and non-stroke cases. The model was trained using the Adam optimizer. This hybrid ViT and biLSTM model combines the temporal capabilities of BiLSTM with the attention capabilities of transformers to automatically identify MRI images that display evidence of stroke. The general workflow of the proposed hybrid ViT–BiLSTM architecture is shown in Figure 2.

In the study, we further developed a sophisticated vision transformer (ViT) architecture, which is known for its ability to process visual data using self-attention techniques. Detecting brain stroke on MRI scans required a very optimized architecture. Using the ViT backbone, our model is capable of extracting hierarchical representations that expose noteworthy patterns suggestive of brain stroke. ViT uses self-attention mechanisms; therefore, it captures long-range dependencies and global context more efficiently than conventional convolutional neural network (CNN) methods. To improve the ViT encoder for this application, we made several modifications. First, we removed the multi-layer perceptron (MLP) layer to increase computational efficiency and included layer normalization to expedite convergence and steady training (21). Unlike CNNs, in which dense layers often add to significant computational needs, this change is different. Subsequently, we added a dropout layer to reduce overfitting and enhance generalization. Adding a “flatten” layer at last advances the model’s ability to discriminate between many visual patterns in the MRI results. Together, these adjustments improve the reliability and efficacy of the model in detecting brain injuries (22). Table 2 lists all the hyperparameters employed in our ViT-BiLSTM system. The parameters for the pre-trained transfer learning model were selected using the same technique. Employing a T4 GPU with 12 GB of RAM, the training process was conducted on Kaggle. To mitigate overfitting, various strategies were employed, such as using dropout and batch normalization for regularization, applying layer normalization within transformer blocks, conducting 5-fold cross-validation, and managing model complexity. Early stopping and adjustments to the learning rate were utilized to ensure stable convergence, with training capped at 20 epochs. Model performance was assessed using metrics such as accuracy, precision, recall, F1-score, as well as receiver operating characteristic (ROC) and PR curves.

**Table 2:** Hyperparameters of the proposed model with their values.

Hyperparameters	Values
Epochs	20
Batch size	16
Image size	224 × 224 × 3
Learning Rate	0.0001
Patch size	16
Optimizer	Adam
Loss function	Categorical Cross-Entropy
Projection dimension	64
Number of patches	196
Number of self-attention heads	4
Number of transformer encoder layers	8

**Figure 3:**
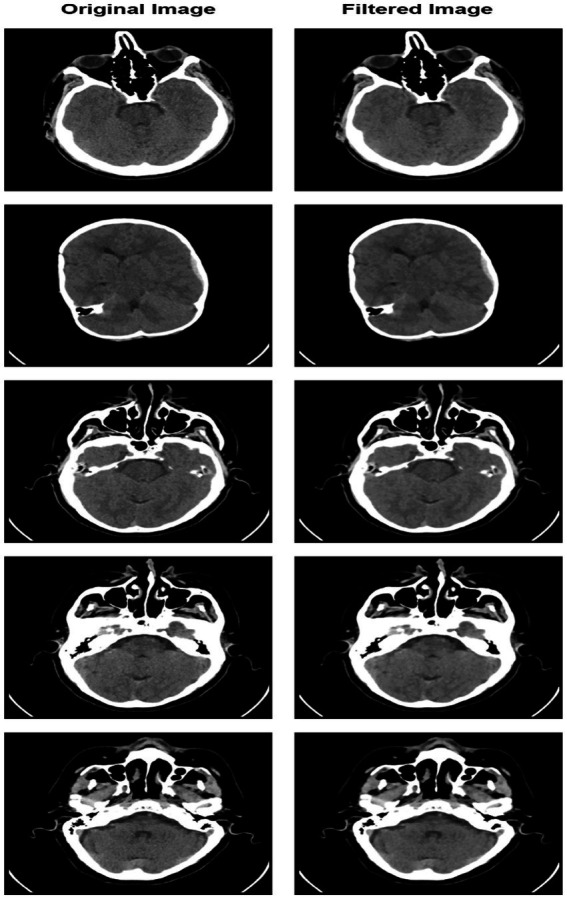
Sample pre-processed image.

The pre-processing steps involve a sequence of operations that apply median filtering techniques to reduce noise and small intensity variations while preserving structural edges in MRI images. Subsequently, the images are resized to 224×224 pixels, as required by the vision transformer framework. Enhancing the accuracy and usefulness of images in illness classification critically depends on pre-processing. Although raw images contain a wealth of information, they can also have flaws that prevent categorization; thus, this deliberate approach was established with an understanding of these limitations. Finally, before training the model, the intensity values of the pixels are normalized for stable convergence and better generalization of the model. A carefully implemented series of thorough pre-processing methods was intended to improve image quality and enable accurate illness categorization. Original and pre-processed images of the specimens are presented in Figure 3.

**Figure 4:**
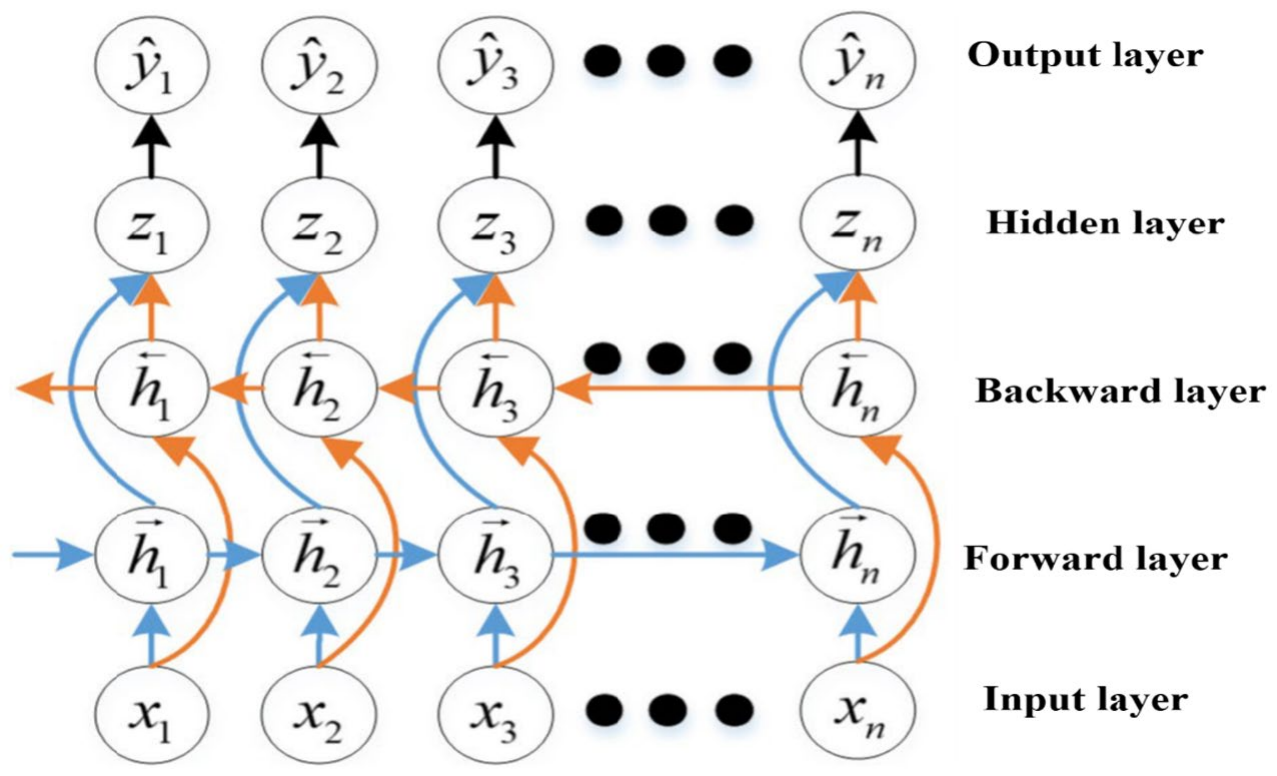
BiLSTM structure.

The BiLSTM is a type of LSTM with an impressive ability to predict complex system behaviors effectively. The BiLSTM consists of two LSTMs: one in the forward direction and one in the reverse direction. Figure 4 shows an overview of how the BiLSTM can understand both the characteristics of sequence data before and after a given time frame. Through bidirectional learning, the BiLSTM improves its non-linear predictive ability, enabling it to provide more accurate predictions than conventional unidirectional LSTMs.

This study uses a three-phase approach to detect brain stroke (BS). The initial phase of data preparation involved the conversion of DICOM images into JPG images, labeling each image according to the stroke it represents, and splitting the images into training, test, and validation datasets. Model development and evaluation involved training and testing a hybrid ViT-BiLSTM model to maximize accuracy, with the evaluation performed on a test dataset. The vision transformer encoder directly used the features it extracted. An additional step in which features were selected separately was not required because the vision transformer encoder extracts features that contextually build on each other through time using Bidirectional long short-term memory, which is very effective at capturing this context. The combined model was also trained in an end-to-end fashion using the Adam optimizer, which provided efficient and stable convergence.

## 4 Results and discussion

A private dataset consisting of MRI scans included axial 2D T2-weighted turbo spin echo (TSE) images obtained with a 1.5 T scanner. The acquisition parameters were configured as follows: repetition time (TR), 4620.0 ms; echo time (TE), 105.0 ms; slice thickness, 5.0 mm; and in-plane resolution, 512 × 512 pixels. Initially, these images were stored in the DICOM format and represented 2D cross-sectional slices rather than 3D volumes. To standardize the input data and improve the computational efficiency of the deep learning pipeline, the 16-bit DICOM files were converted into 8-bit JPEG files. Although this conversion reduces the original intensity depth, the essential morphological features and contrast differences necessary for stroke lesion classification are clearly preserved.

**Figure 5:**
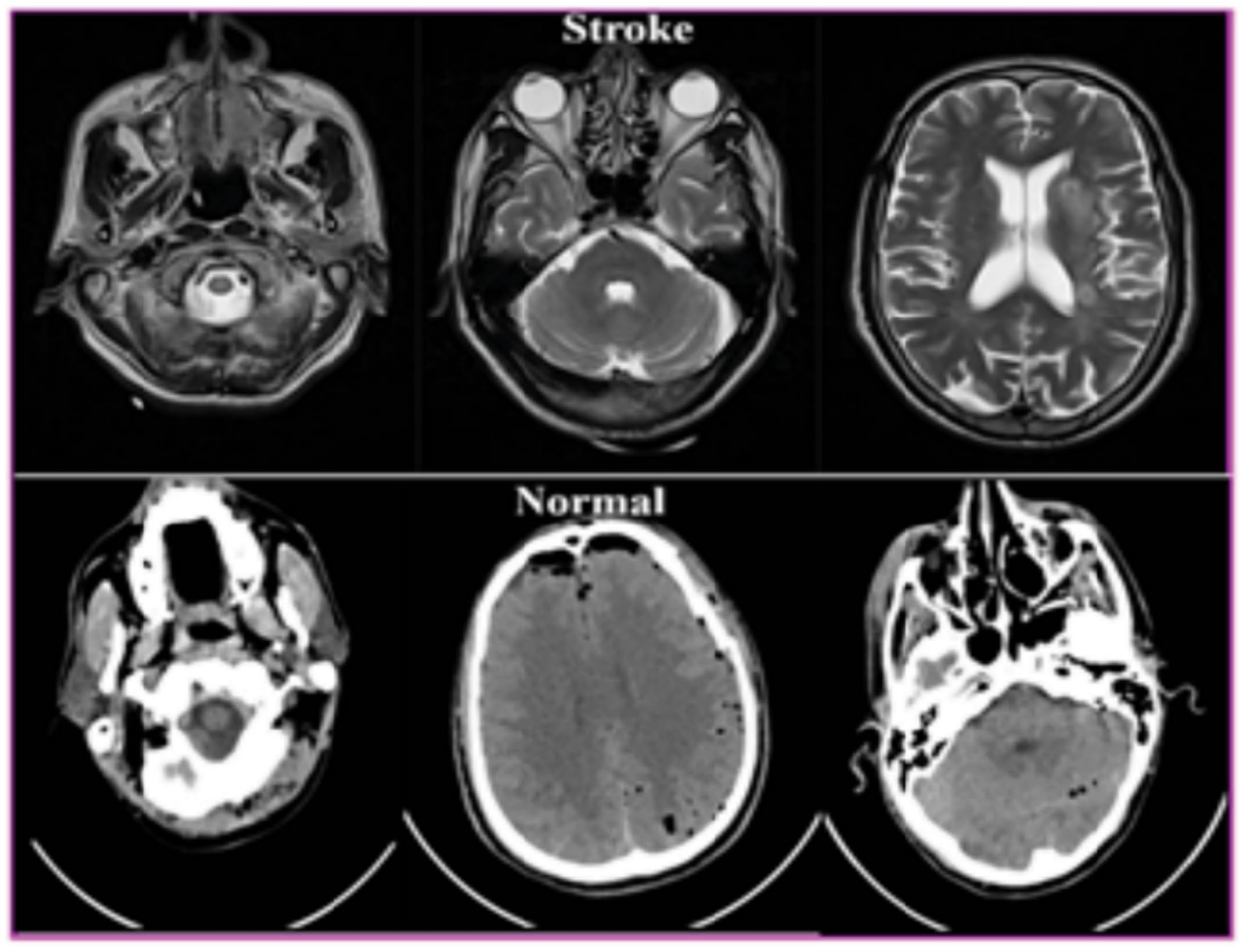
Sample images of the brain stroke dataset.

The dataset consisted of MRI images that were part of the surgical planning for brain stroke treatment and included clinical data with a unique patient identification number. The study conducted in this paper used a private brain stroke dataset containing 3,125 MRI images of stroke for stroke classification. The dataset was divided into two categories: 1551 normal images and 1,574 stroke images. The images were separated into three groups, with 70% of the images allocated to training, 20% of the images allocated to validation, and 10% of the images allocated for testing. Table 3 shows the distribution of the brain stroke dataset. Once the input images were collected, the output images were classified and annotated as stroke or normal images, depending on the presence or absence of stroke depicted in the image. A sample of the prepared data is shown in Figure 5. We confirmed that all methods and experiments conducted were purely computational and did not involve any human subjects directly. Patient details were highly confidential.

- We confirm that all methods were performed in accordance with relevant guidelines and regulations.
- We confirm that informed consent was obtained from all participants and/or their legal guardians.

**Table 3:** Analysis of brain stroke dataset.

Dataset	Total images	Training set	Testing set	Validation set
Brain stroke	3,125 (Normal–1,551, Stroke −1,574)	2,249	313	563

A comprehensive comparison of several deep learning models evaluated for the purpose of categorizing brain strokes is presented in Table 4. The baseline CNN model performed moderately well, with an AUC of 88.30% and an accuracy of 85.50%. Deeper convolutional layers caused a slight improvement in both ResNet50 and VGG16. The vision transformer (ViT) demonstrated excellent discriminatory capabilities, achieving an accuracy of 91.30% and an AUC of 93.20%. BiLSTM, which is well-known for its ability to identify sequential dependencies, performed better in terms of recall and F1-score. The proposed hybrid ViT-BiLSTM model performed remarkably well compared to other models, achieving an accuracy of 95.21%, a precision of 97.35%, and an outstanding AUC of 99.36% across all metrics. The efficacy of integrating spatial and temporal feature extraction for a reliable stroke diagnosis was demonstrated by these results.

**Table 4:** Evaluation of model performance.

Model	Acc (%)	Prec (%)	Recall (%)	F1 Score (%)	AUC (%)
CNN (Baseline)	85.50	84.20	86.10	85.10	88.30
ResNet50	89.20	88.50	89.80	89.10	91.50
VGG16	87.80	86.90	88.20	87.50	89.70
Vision Transformer (ViT)	91.30	90.80	91.70	91.20	93.20
BiLSTM	88.60	87.40	89.10	88.20	90.40
Hybrid ViT-BiLSTM (Proposed)	95.21	97.35	93.04	95.15	99.36

**Figure 6:**
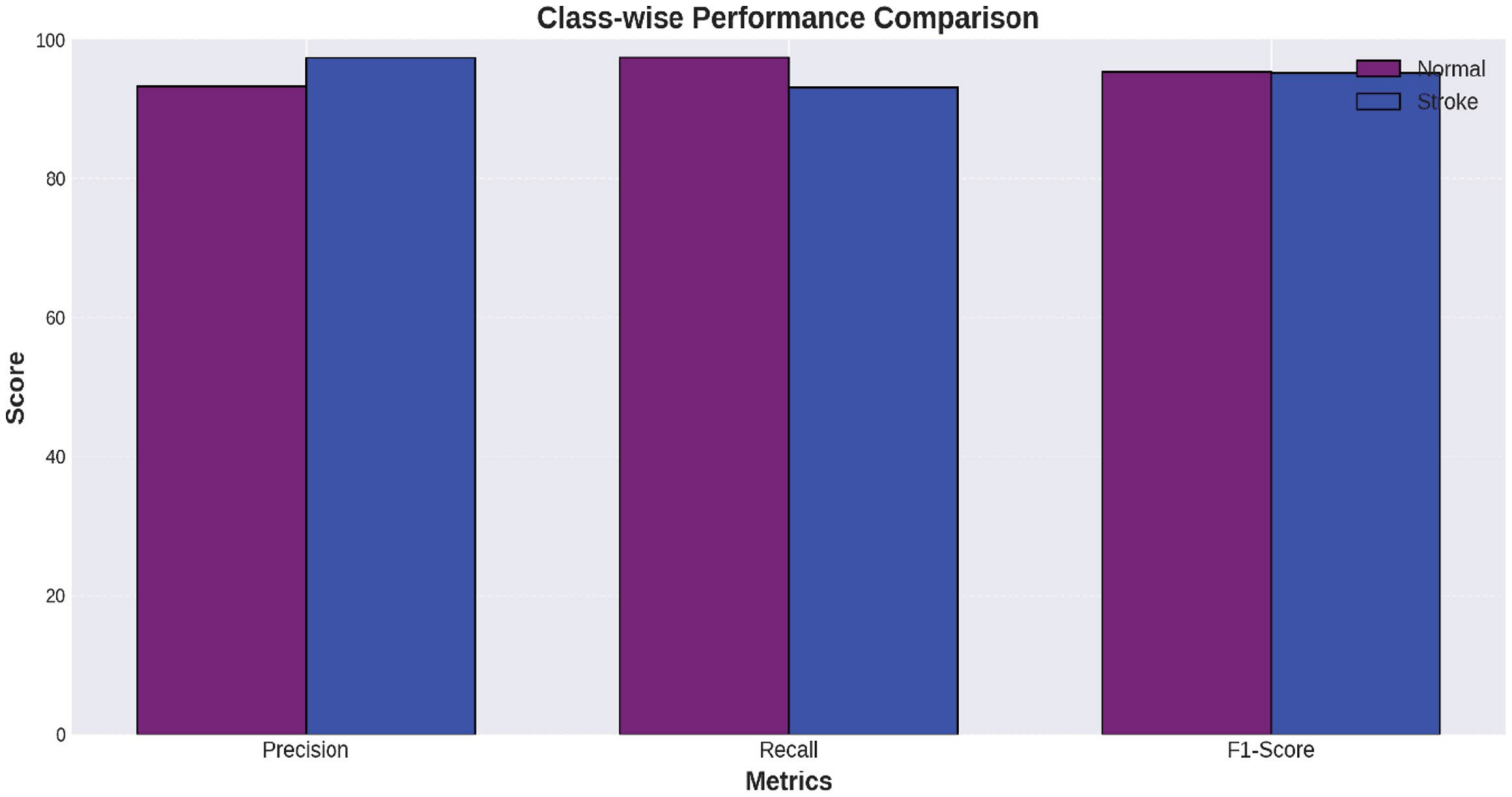
Class-wise performance metrics.

**Figure 7:**
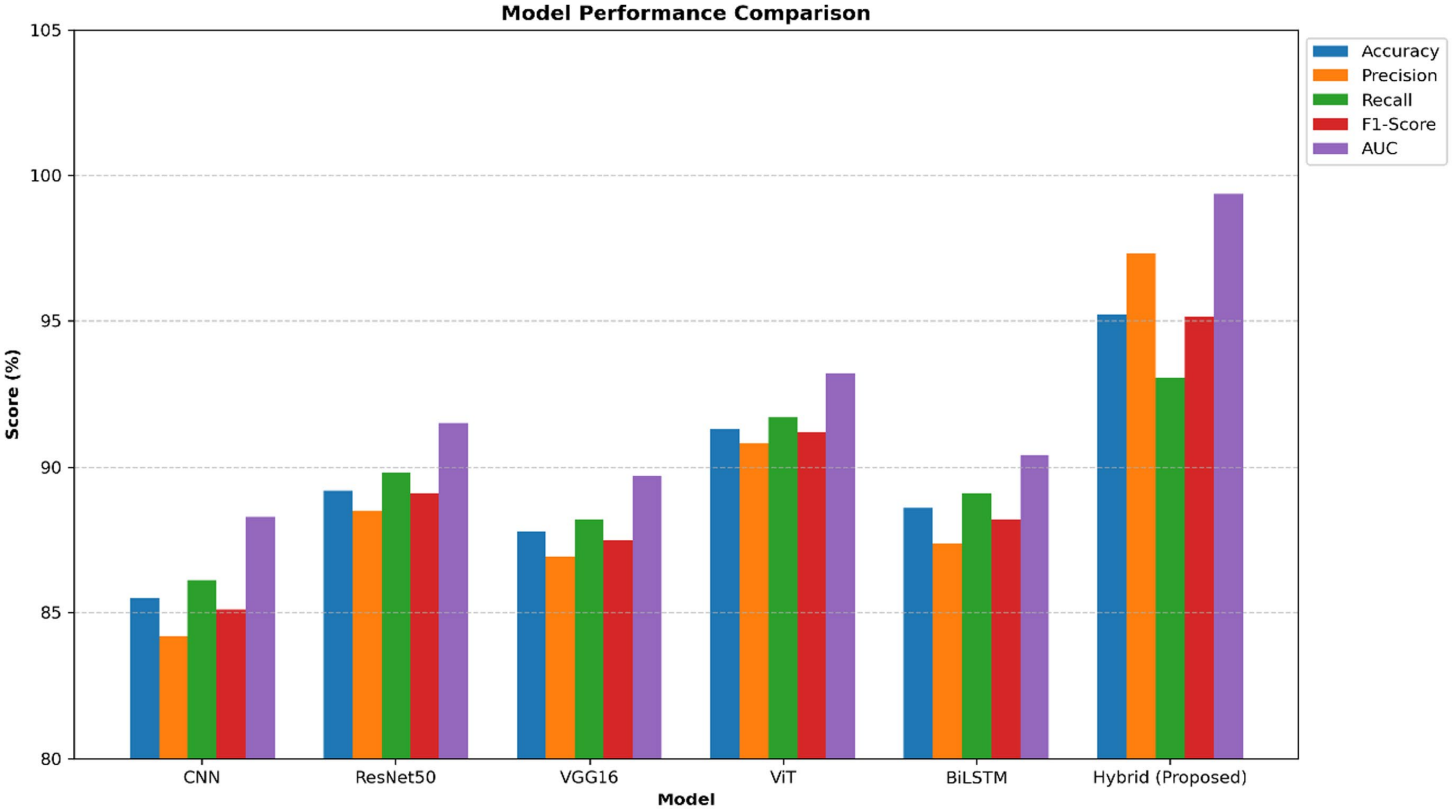
Model performance analysis.

**Figure 8:**
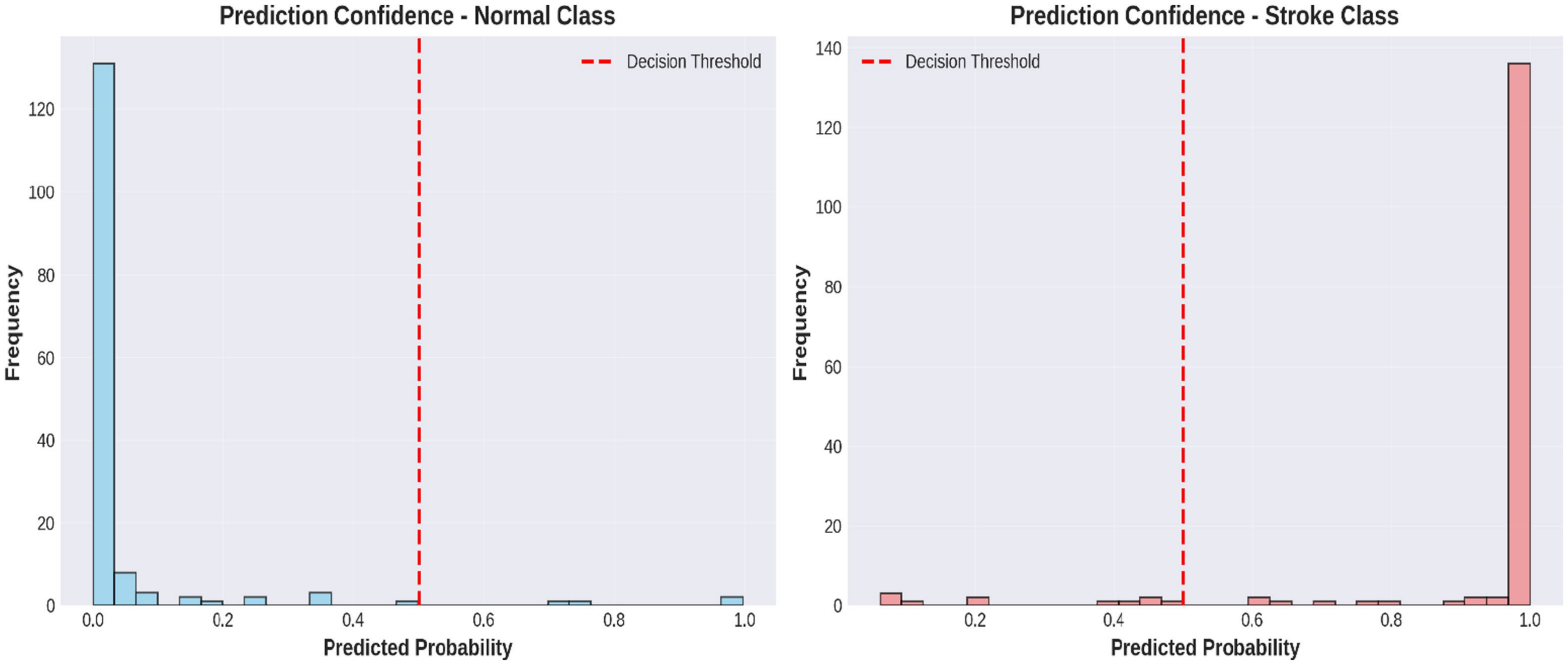
Prediction confidence distribution for normal and stroke classes.

Figure 6 shows a performance comparison between normal and stroke classes. Figure 7 shows a comparison of the model performances. Figure 8 shows that the proposed Hybrid ViT-BiLSTM model delivers impressive performance and stability, as evidenced by smooth training convergence over 20 epochs, during which the accuracy and AUC improved, whereas the loss decreased. The model exhibited outstanding classification reliability, as demonstrated by a confusion matrix with only 15 errors out of 313 samples and prediction confidence histograms, where most probabilities were concentrated near the extremes of 0.0 and 1.0. Additionally, the nearly flawless paths of the ROC (AUC = 0.9936) and precision-recall (AUC = 0.9937) curves validated the strong capability of the architecture to differentiate between classes with high precision and sensitivity.

**Figure 9:**
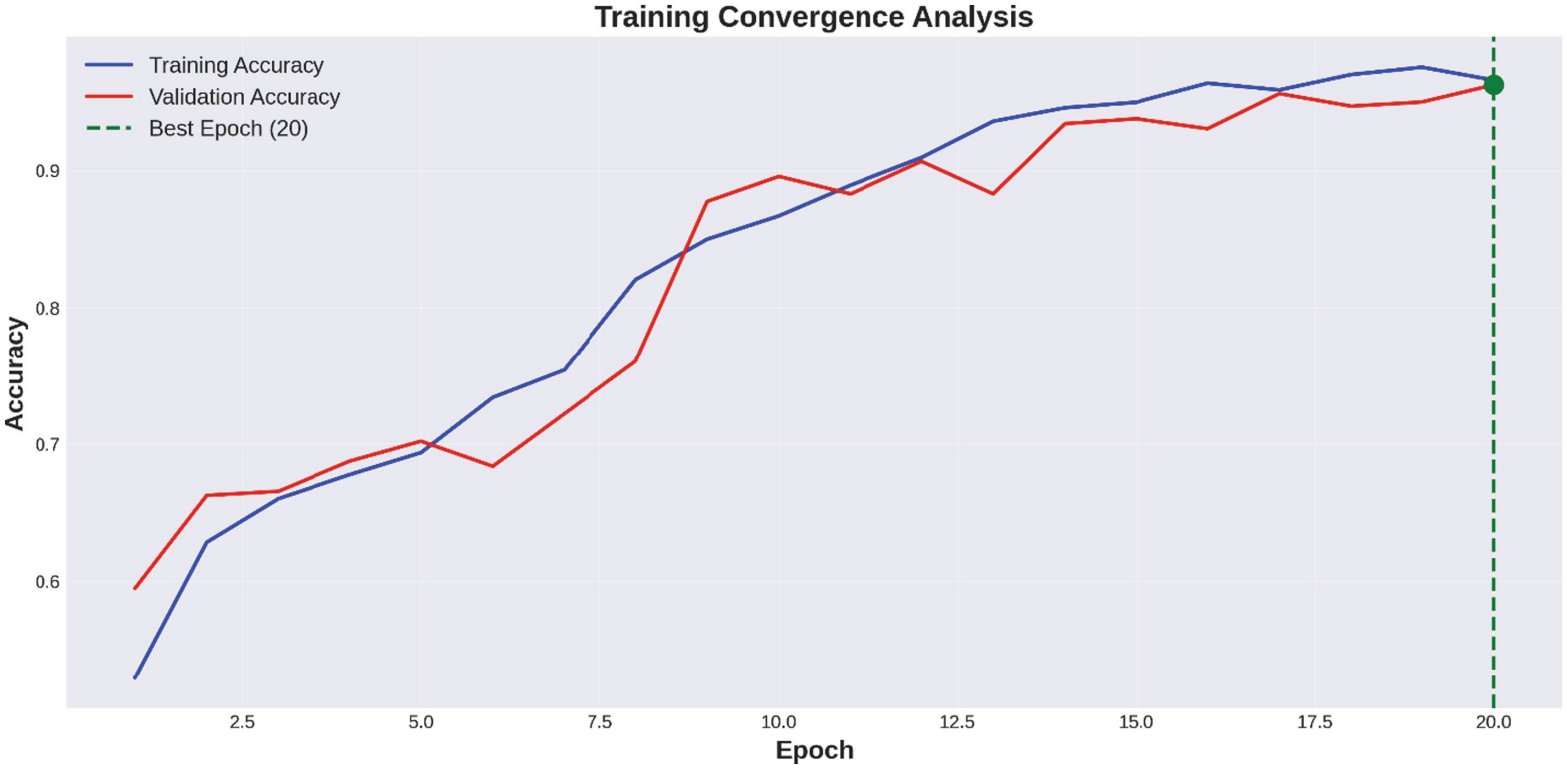
Convergence analysis.

**Figure 10:**
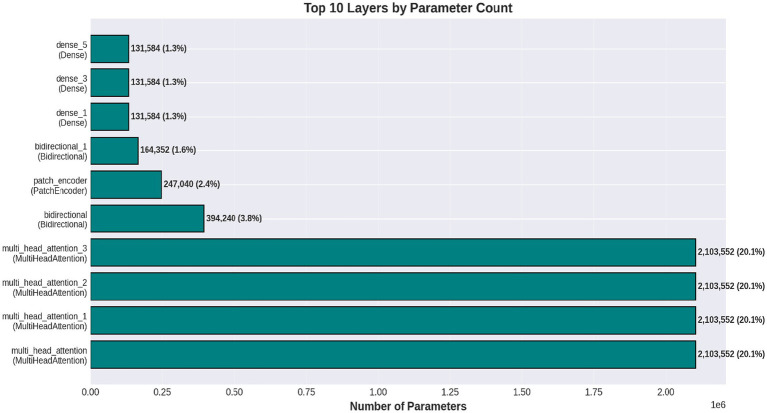
Model complexity.

**Figure 11:**
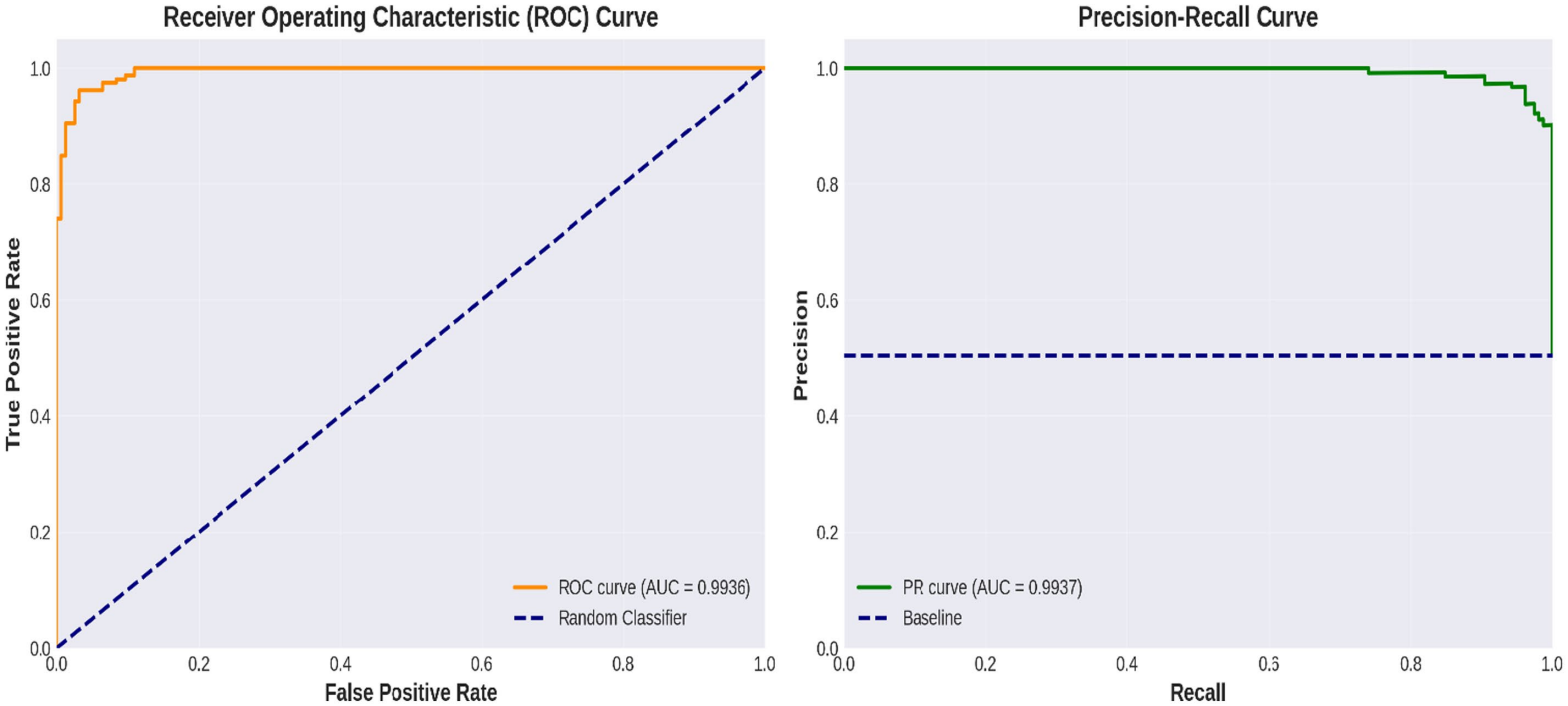
ROC-PR curves.

**Figure 12:**
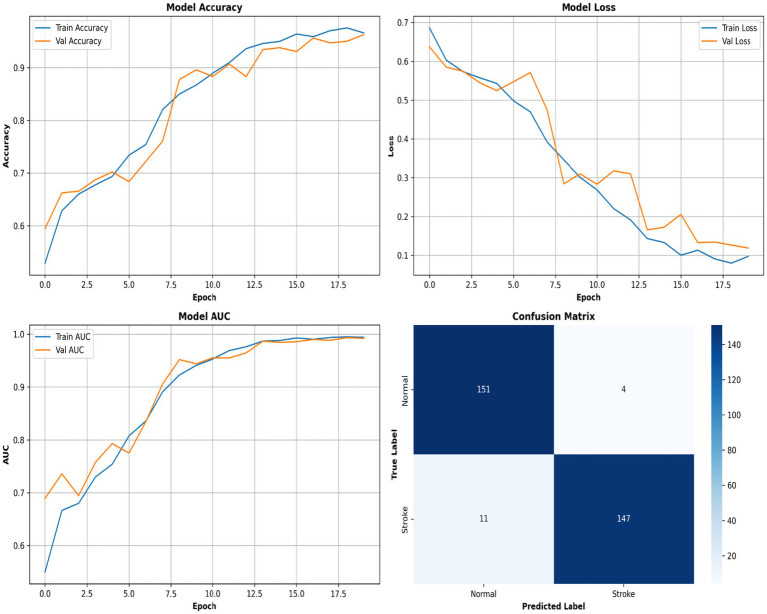
Comprehensive model evaluation across epochs.

**Figure 13:**
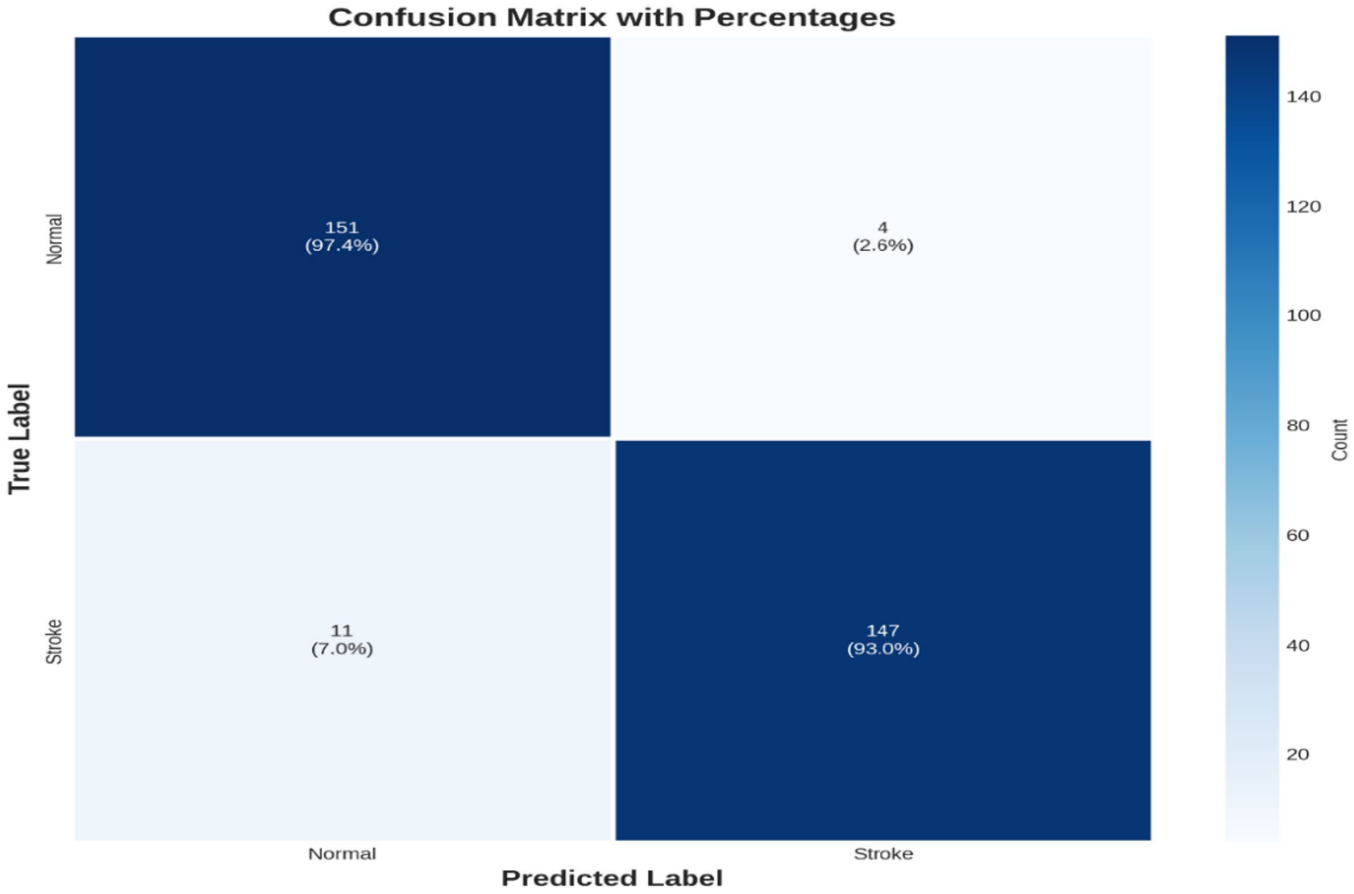
Normalized confusion matrix.

Figure 9 depicts the progression of the model accuracy across 20 epochs. The blue and red lines represent the training and validation accuracies, respectively, reaching their highest points at epoch 20, as indicated by the green dashed and dotted lines. This signifies the ideal training stage, in which performance and generalization are optimally balanced. Figure 10 illustrates the complexity of the model in terms of the number of parameters. The Hybrid ViT-BiLSTM model outperformed the baseline CNN and ViT models with an accuracy of 95.21% and an AUC of 0.9936. Even with full recall, the ROC and PR curves showed almost perfect trajectories, indicating class distinction and high precision. These results confirmed the model’s resilience and efficacy in addressing intricate categorization complications. The ROC and PR curves are shown in Figure 11, and the performance metrics of the Hybrid ViT-BiLSTM model are shown in Figure 12. This covers the training process and emphasizes the confusion matrix, accuracy, loss, and AUC. Figure 13 shows the normalized confusion matrix, which illustrates that the model accurately identified 97.4% of normal cases and 93.0% of stroke cases, reflecting strong performance for each class. The rates of misclassification were minimal, with only 2.6% of normal samples and 7.0% of stroke samples being incorrectly predicted, highlighting the model’s high sensitivity and specificity. Despite the limited number of instances in the dataset, several methods were used to prevent overfitting in the model. This is evident in the use of the 5-fold cross-validation method to validate the performance of the model, which showed that the model performed stably with minimum variance.

## 5 Ablation study

Table 5 illustrates the ablation study conducted to assess the impact of each component within the proposed model. Among the baseline models, the CNN achieved an accuracy rate of 85.50%, whereas ResNet50 and VGG16 enhanced the performance to 89.20 and 87.80%, respectively. In the ablation setups, the standalone ViT model attained an accuracy of 91.30%, highlighting the effectiveness of transformer-based feature extraction, whereas the standalone BiLSTM achieved an accuracy of 88.60%. The CNN–BiLSTM configuration slightly boosted the performance to 89.71%, suggesting the advantage of integrating spatial and temporal feature learning. Nevertheless, the proposed hybrid ViT–BiLSTM model significantly surpassed all baseline and ablation configurations, attaining the highest accuracy of 95.21%, precision of 97.35%, recall of 93.04%, F1-score of 95.15%, and AUC of 99.36%. Compared to the best-performing ablation component (ViT-only), the proposed model improved accuracy by 3.91%, precision by 6.55%, recall by 1.34%, F1-score by 3.95%, and AUC by 6.16%. These findings demonstrate that the combination of a vision transformer with a BiLSTM effectively progresses feature representation and enhances classification performance for brain stroke detection.

**Table 5:** Ablation study results: component-wise performance analysis.

Configuration	Acc (%)	Prec (%)	Rec (%)	F1 (%)	AUC (%)	Params	Infer. Time (ms)
Baseline models							
CNN (Baseline)	85.50	84.20	86.10	85.10	88.30	0.5 M	12
ResNet50	89.20	88.50	89.80	89.10	91.50	25.6 M	38
VGG16	87.80	86.90	88.20	87.50	89.70	138 M	45
Ablation configuration							
ViT-Only	91.30	90.80	91.70	91.20	93.20	2.1 M	95
BiLSTM-Only	88.60	87.40	89.10	88.20	90.40	0.7 M	45
CNN-BiLSTM	89.71	88.90	90.30	89.60	91.80	0.8 M	68
Hybrid ViT-BiLSTM	95.21	97.35	93.04	95.15	99.36	2.8 M	150
Improvement over the best ablation component							
vs. ViT-Only (a)	+3.91%	+6.55%	+1.34%	+3.95%	+6.16%	–	–
vs. BiLSTM-Only (b)	+6.61%	+9.95%	+3.94%	+6.95%	+8.96%	–	–
vs. CNN-BiLSTM (c)	+5.50%	+8.45%	+2.74%	+5.55%	+7.56%	–	–

**Figure 14:**
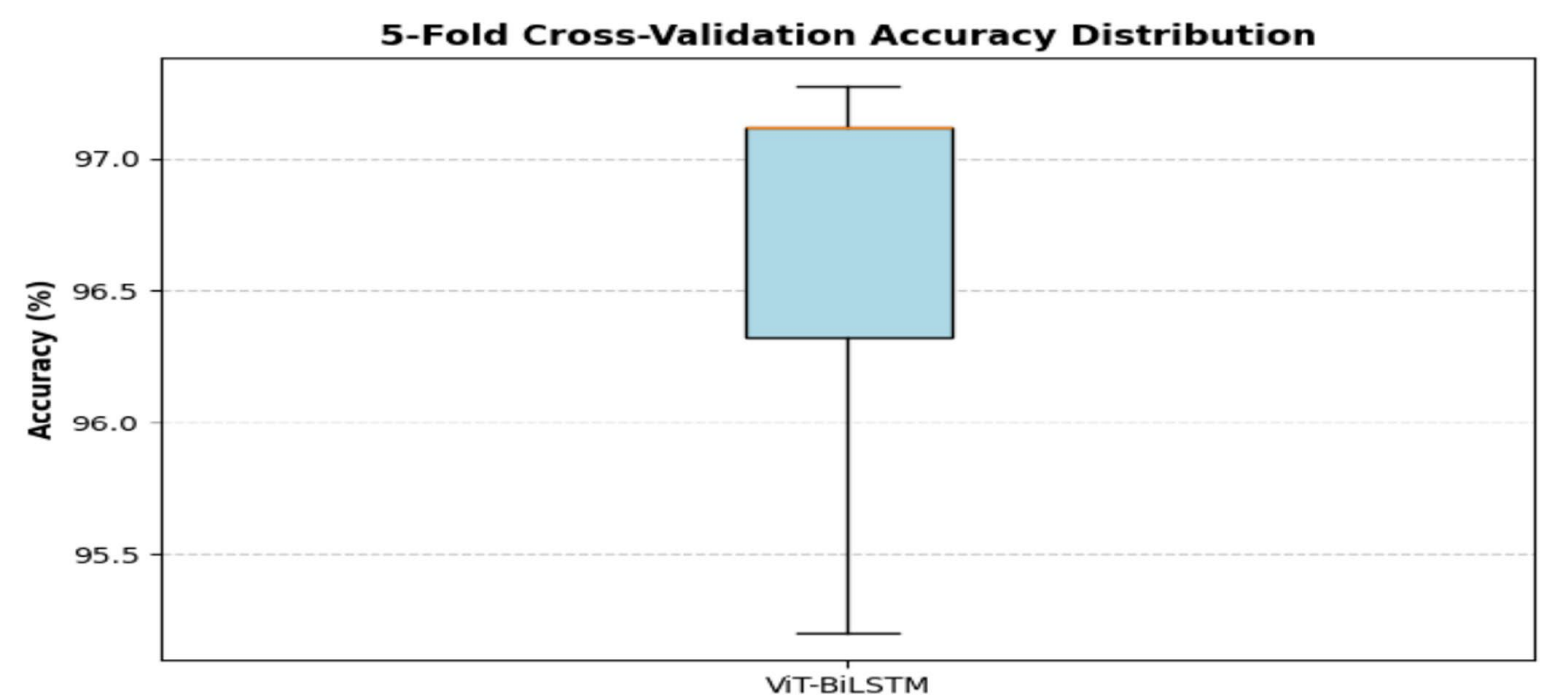
5-fold cross-validation.

Table 6 presents the results of the 5-fold cross-validation for various configurations. The hybrid ViT-BiLSTM model proposed in this study achieved the highest average accuracy of 96.61%, with a low standard deviation of 0.78, reflecting its stable and consistent performance across different folds. This hybrid architecture outperformed other configurations in terms of generalization capability. Figure 14 shows the accuracy distribution obtained from the 5-fold cross-validation for the ViT-BiLSTM model.

**Table 6:** Cross-validation results (5-fold).

Configuration	Mean Acc (%)	Std Dev	Min	Max
ViT-Only	91.12	1.34	89.45	92.78
BiLSTM-Only	88.42	2.67	85.23	91.04
CNN-BiLSTM	89.58	1.89	87.34	91.52
Hybrid ViT-BiLSTM	96.61	0.78	95.20	97.12

## 6 Conclusion

This study provides a deep learning methodology that integrates a vision transformer with a bidirectional long short-term memory network for classifying brain strokes in MRI scans. The vision transformer captures and processes both global spatial and contextual information through self-attention mechanisms, whereas the bidirectional long short-term memory network captures and models bidirectional temporal dependencies on data embeddings of those spatial and contextual features. The combination of these models enables the proposed model to learn unique spatial features and their relationships to other spatial and contextual features, thus improving the accuracy of stroke classification.

The hybrid ViT–BiLSTM model, implemented with the Adam optimizer, demonstrated a stable convergence rate during training. This model achieved the following: Querying results showed a precision of 97.35%, recall of 93.04%, accuracy of 95.21%, F1 score of 95.15%, and ROC-AUC of 99.36%, which is higher than that of most comparable methods. The proposed model exhibited excellent generalization abilities, reaching an average accuracy of 96.61% during 5-fold cross-validation. The small standard deviation of 0.78 suggests that its performance was stable and consistent across various folds. When using only the vision transformer, the accuracy was 91.3%, which is higher than that of traditional CNN-based methods. Thus, these results provide evidence of the benefits of incorporating bidirectional temporal modeling along with transformer-based feature extraction methods.

In addition, the proposed ViT-BiLSTM framework demonstrated an exceptional level of performance and stable generalization across a variety of datasets and can provide automated diagnosis of brain stroke in clinical applications.

### 6.1 Limitations of the study

- The dataset used in this study was relatively small and may not fully capture variations in stroke characteristics, imaging protocols, scanner types, and patient demographics, which may affect the generalizability of the model.
- DICOM MRI images were converted to JPEG images. Although this aids computation, minor information loss during conversion could affect fine features
- Splitting in the given dataset has not been done based on patient identifiers; however, image splitting has been performed. This may cause some potential data leakage, which could lead to an optimistic estimation in the future.
- The data were sourced from a single medical institution, and the lack of multicenter data may impact the robustness of the model when applied to images from different institutions and equipment.
- External validation using independent datasets was not conducted. Although the model performed well in internal testing, validating it on public or multi-institutional datasets would enhance confidence in its generalization capabilities.
- The hybrid architecture includes 10.4 million trainable parameters, thereby increasing the computational complexity. The self-attention mechanism in the vision transformer and the BiLSTM sequential processing demand significant resources, which poses a challenge for deployment in resource-limited settings.
- The framework does not utilize explainability techniques, such as Grad-CAM or SHAP, to offer visual or feature-level insights into model decisions, which may hinder clinical transparency and trust.

### 6.2 Future work

- Future work will focus on validating the proposed model using larger external MRI datasets to evaluate its generalization capability and robustness, given the limited dataset size used in this study.
- The incorporation of FL provides the advantage of decentralized training while maintaining patient data privacy, which is critical for medical applications, in which patient confidentiality is important.
- Future research should continue to expand this framework to include multicentric datasets and multiple types of neurological disorders.
- Promote intelligent, privacy-preserving diagnostic systems for brain stroke detection, to advance developments in medical imaging and ensure accurate healthcare.
- Future efforts will focus on multicenter validation, optimizing the model for computational efficiency, and incorporating explainable AI techniques to improve its clinical applicability.

## Data Availability

The raw data supporting the conclusions of this article will be made available by the authors, without undue reservation.
